# Influence of the In-Stream Structure on Solute Transport in the Hyporheic Zone

**DOI:** 10.3390/ijerph19105856

**Published:** 2022-05-11

**Authors:** Han Li, Ying Liu, Jinghong Feng, Defu Liu, Yi Li, Lihui Chen, Jingwen Xiao

**Affiliations:** Hubei Key Laboratory of Ecological Restoration of River-Lakes and Algal Utilization, Innovation Demonstration Base of Ecological Environment Geotechnical and Ecological Restoration of Rivers and Lakes, Hubei University of Technology, Wuhan 430068, China; lh10181996@163.com (H.L.); 20140053@hbut.edu.cn (J.F.); liudf@hbut.edu.cn (D.L.); liyi_bnuphd@mail.bnu.edu.cn (Y.L.); clh0710@163.com (L.C.); dream100.0@foxmail.com (J.X.)

**Keywords:** hyporheic zone, in-stream structures, solute transport, numerical modeling, hyporheic change

## Abstract

The hyporheic zone (HZ) plays an important role in the river ecosystem, and hyporheic exchange and solute transport in the HZ are important ecological functions. However, the relationship between the design parameters of river structure and solute transport is still poorly understood. In this study, we combined flume experiments and numerical simulations to systematically evaluate how in-stream structures impact the solute transport depth (D_P_), hyporheic vertical exchange flux (Q), and solute flux (Qs). The results showed that the in-stream structure had a significant influence on solute transport in the HZ and could obviously increase the intensity of hyporheic exchange and promote solute transport. Model results indicated that D_P_, Q, and Qs increased with the ratio of ground height to underground height of structure (*H*/*D*) and structure number (*N*), while Q, D_P_, and Qs increased with the structural spacing (*S*) to begin with; then, Q remained constant, and D_P_ and Qs decreased as *S* continued to increase. This study deepened our understanding of the influence of in-stream structural design parameters on HZ solute transport, which is helpful to provide a theoretical basis for ecological restoration projects in the river HZ.

## 1. Introduction

The hyporheic zone (HZ) is an area under or beside the riverbed, which is characterized by the active mixing of shallow groundwater and surface water, and it is an active and connecting ecotone between groundwater and surface water [1,2,3,4,5,6]. As the interface between surface water and groundwater, the physical, chemical, and biological characteristics of HZ are complex, which is the result of strong mixing of groundwater and surface water [7,8,9]. The hyporheic exchange between the HZ and surface water has a significant impact on the water quality and ecology of the river ecosystem. It could promote heat exchange, controlling the temperature pattern [10,11], affecting the fate and transport characteristics of different solutes within the streambed and altering the residence time of the solutes [12,13,14]. It could also affect the distribution and abundance of organisms in streams and the HZ and the process at the ecosystem level, forming a unique environment of large invertebrates and biogeochemical reactions [15]. If the hyporheic exchange transfers dissolved oxygen from the overlying water to the gravel riverbed, it provides good conditions for the survival and hatching of salmon eggs [2,16]. Therefore, hyporheic exchange has a significant impact on river ecological function and water quality [17,18,19].

Given the importance of the HZ, the ecological restoration of the HZ has also received more attention in recent years [1,20,21,22]. Though the in-stream structures, such as steps, pools, and wooden dams, have often been used for ecological restoration of river HZ for a long time, it was proved that these structures will affect the water head gradient along the sediment–water interface (SWI) recently, thus driving hyporheic circulation with different lateral ranges and penetration depths [23].

In recent years, a large number of field experiments and indoor simulation studies have shown that the in-stream structure in the river has a significant impact on hyporheic exchange. In the study of Mutz et al. [24], the indoor circulating flume experiment was used to change the riverbed topography by adding wood to form the internal structure of the riverbed. The results showed that the flow resistance increased by 2-fold, and the vertical water flux through the riverbed increased by 1.8–2.5-fold. In the study of Smidt et al. [25], the hyporheic exchange caused by the stream restoration structure (horizontal leaf) and natural geomorphology (gully) was compared by using the delay resistivity (ER) tomography technique, and the results showed that the repaired structure may be able to create sufficient exchange flow and transport time to achieve the same ecological functions as natural characteristics. Based on the coupling of a large flume sediment transport experiment and computational fluid dynamics, the effect of river shape change caused by pebbles on the underflow was studied. The results showed that placing pebbles on a plane bed could increase the median residence time of the underflow on the river scale by 15% and the downstream flux by 18% [26]. Rana et al. [27] constructed a series of weirs in small rivers to simulate natural debris dams, river restoration log dams, pebble dams, and other structures spanning the channel. Additionally, they then conducted constant-rate conservative (NaCl) tracer injection experiments using transient storage of a one-dimensional solute transport model to quantify the change in solute transport parameters with an increasing number of weirs. The results showed that the addition of weirs significantly increased the surface water flow (A) and the cross-sectional area of the transient storage area (As). The numerical simulation study of Hester et al. [28] showed that reach-scale NO_3_^−^ removal from in-stream structures and inset floodplains is highly sensitive to changes in sediment conditions, biogeochemical parameters, and design parameters. Wade et al. [29] researched an artificially constructed permeable structure to promote hydraulic head differentials to induce exchanges and observed it provided higher vertical fluxes, zones of spatially varying nitrate production and anaerobic reduction, and found that exchanges were enhanced only after a certain structure height was crossed and that it had little influence on surface water chemistry.

Other literature studies have also focused on different structural designs to study the effects of structures on hyporheic exchange. Hester and Doyle [30] used numerical simulations to study the effect of three structure types (weirs, steps, and lateral structures) on subsurface flow exchange. The results showed that the structure types are different in their ability to induce hyporheic flow; channel crossing structures (weir and step) are usually more effective than partial crossing structures (lateral structures), and weirs are more effective than steps. Ward et al. [31] analyzed the designed structure length, structure height, and hydraulic conductivity and concluded that the hyporheic flux could be potentially changed by hyporheic restoration structures. In the work of Liu and Chui [6], based on numerical simulation for different weir heights, model data were used to establish the regression equation of related variables; the denitrification effect of the HZ was comprehensively considered, and the best weir height could be obtained.

The purpose of these studies can be summarized into these two aspects: (1) the optimal design of the structure and (2) the HZ function under the influence of the structure. However, most of the research focuses on the possible changes of HZ caused by the structure in the river, without analyzing the causes of the changes by different parameters of the structure. Nowadays, there is still a lack of research on the exchange of subsurface flow caused by in-stream structures, especially the influence of the ratio of ground height to underground height on structure (*H*/*D*) and spatial positions. Therefore, the ways of repairing the river hyporheic zone are mostly empirical.

To fill this gap, numerical simulations were used to understand how design parameters of the in-stream structure affect hyporheic exchange in this study. Weir was selected as the representative river structure because it was considered to be the most effective method to improve the hyporheic flux [30]. The specific objective of this paper is to research the effects of structure proportion change, spacing change, and number change on HZ solute transport, such as solute transport depth D_p_, vertical hyporheic exchange flux Q, and solute flux Qs. The research will enhance our understanding of the in-stream structures that induce HZ performance, which could be of benefit for the hyporheic zone restoration.

## 2. Methods

### 2.1. Laboratory Experiments

#### 2.1.1. Flume Setup

**Figure 1:**
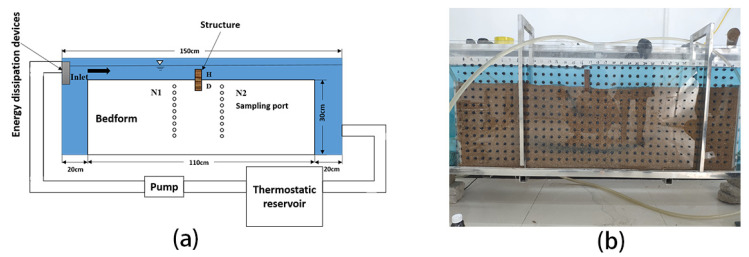
Schematic diagram (**a**) and actual diagram (**b**) of experimental apparatus. The light gray and blue areas represent the parts filled with sand and water, respectively. The dark gray rectangle on the sand bed is the weir set in the experiment. N1 and N2 are two rows of sampling holes with a distance of 5 cm to the structure.

In this experiment, we used an indoor constant temperature circulating flume. Referring to the flume test method of Jin et al. [32], the length, height, and width of the flume were 1.5 m, 0.5 m, and 0.1 m, respectively, as shown in Figure 1. To facilitate the observation of the experiment, we used 1 cm thick acrylic acid to build the tank wall. An energy dissipation device was installed at the inlet of the flume to reduce or eliminate the tumbling and fluctuation of the flow at the inlet to make the flow into the riverbed more stable. The flow velocity of the flume was 0.04 m·s^−1^. The surface water temperature of the tank is controlled by the heating/cooling system connected to the additional water recycling side loop. Water from the side loop is mixed with the main loop. Then, the surface water flow is controlled by the valve and measured by the electromagnetic flowmeter. To avoid influence in the sampling process, we arranged sampling holes on the side of the flume to facilitate sampling.

#### 2.1.2. Bed Sand Properties and Preparation

In the experiment, sand or gravel with sifted 0.25 mm~0.5 mm particle sizes were selected as the filler to shape the riverbed. In this experiment, the effective sand bed length was 1.1 m, and the thickness was 0.3 m. Before the experiment, we washed the sand with tap water three times to remove impurities and organic matter. To represent a channel-spanning weir, a 9 cm tall channel-spanning weir was placed at the channel center. The buried depth *D* of the structure was 5 cm, and the height *H* of the structure on the water interface was 4 cm.

#### 2.1.3. Experimental Conditions and Measurements

Before the start of the experiment, we filled the flume with tap water, and then, a sand bed was laid, ensuring that it was fully saturated. The customized plank was inserted into the middle part of the sand bed, and its embedding depth was strictly controlled according to the industrial and mining settings. After that, the flume was debugged, and the constant temperature water tank was opened to ensure that the water temperature was kept at a constant 20 degrees Celsius during operation.

We selected NaCl as the nonreactive tracer in this experiment. The weighed sodium chloride was stirred and dissolved completely and then slowly added to the circulating water tank, stirring evenly to ensure that the initial concentration of sodium chloride in the tank was 2.3 g/L. To monitor the process of solute transport in the riverbed, pore water samples were collected from the sampling mouth over a period of time. A 100 microliter syringe with a fine needle was used to sample through a plastic spool on the wall of the sink. One hundred microliters of pore water was extracted from each sample. The relatively small sampling volume is unlikely to significantly affect the flow field near the sampling port. At the beginning of the experiment, the sampling interval of pore water was 30 min to capture the relatively rapid change in solute concentration when it entered the bed. With the progress of the experiment, the change in concentration tended to be smooth after the local solute front passed through. The sampling interval was increased to a few hours near the end of the experiment. The water sample was diluted with 5 mL of deionized water to obtain a sufficient volume for subsequent electrical conductivity (EC) measurements (using a Swiss-made Mettler Toledo S230).

To compare and analyze the migration of the concentration tracer in the hyporheic zone under different positions and different working conditions, the tracer concentration was normalized in this experiment. The ratio of the tracer concentration in the monitoring hole to the initial concentration of the tracer was defined as a dimensionless relative concentration, C/C_0_, where C is the sample concentration, and C_0_ is the initial concentration of surface water.

### 2.2. Numerical Simulations

**Figure 2:**
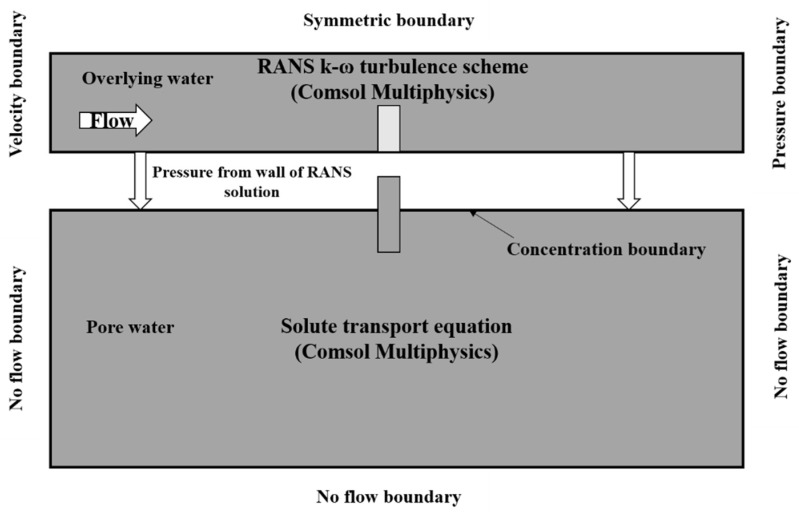
Schematic of the simulation domain and boundaries. Top part shows the model boundary conditions for the turbulent flow model. Bottom part illustrates boundary conditions for groundwater flow and solute transport models.

In this paper, we used COMSOL Multiphysics numerical simulation software (Shanghai, China) to build a two-dimensional river surface water–groundwater coupling model and used the one-way sequential coupling method to numerically simulate the flow, pore water, and solute transport in the riverbed. First, a numerical simulation of the surface water flow was carried out, and the pressure distribution of the SWI was obtained. On this basis, the pore water flow field driven by the pressure distribution was simulated, and the solute transport situation was obtained. This approach largely follows that of Cardenas and Wilson [33]. The basic conceptual model is shown in Figure 2.

#### 2.2.1. Mathematical Model and Boundary Conditions for Overlying Water

In this study, we assumed that the fluid was homogeneous and incompressible, and that the bed base was homogeneous, isotropic, and free of any displacement. The boundary conditions of the model are shown in Figure 2. The left boundary was the inlet velocity boundary, the right boundary was the pressure outlet boundary, the upper boundary was set as the symmetrical boundary, and the lower boundary was the wall boundary, which was set to no flow. The flow is governed by the Reynolds-averaged Navier–Stokes (RANS) and the *k*-*ω* turbulence model. For incompressible fluids, the steady-state RANS equation is defined as

(1) $$\frac{\partial \rho U_{i}}{\partial x_{i}}=0\;$$

(2) $$\rho \frac{\partial U_{i}}{\partial t}+\frac{\partial \rho U_{i}U_{j}}{\partial x_{j}}=-\frac{\partial P}{\partial x_{i}}+\frac{\partial }{\partial x_{j}}\left(2\mu S_{ij}-\rho \bar{{u^{\prime }}_{i}{u^{\prime }}_{j}}\right)\;$$

where *ρ* and μ refer to the fluid density and dynamic viscosity (hypothetical standards for water), respectively, $U_{i}\;\mathrm{or}\;U_{j}$ (*i*, *j* = 1, 2, where *i* ≠ *j*) refers to the time-averaged velocity, and ${u^{\prime }}_{i}$ refers to the fluctuations in the instantaneous velocity components in *x_i_* or *x_j_* (*i*, *j* = 1, 2, where *i* ≠ *j*). *P* refers to the time-averaged pressure. The strain rate tensor (*S_ij_*) is defined as

(3) $$S_{ij}=\frac{1}{2}\left[\frac{\partial U_{i}}{\partial x_{j}}+\frac{\partial U_{j}}{\partial x_{i}}\right]\;$$

The Reynolds stresses are related to the turbulent kinetic energy (*k*) and specific dissipation rate (*ω*) by

(4) $$\tau_{ij}=-\bar{{u^{\prime }}_{i}{u^{\prime }}_{j}}=\upsilon_{t}\left(2S_{ij}\right)-\frac{2}{3}\delta_{ij}k$$

where $\upsilon_{t}\;$refers to the kinematic eddy viscosity, $\delta_{ij}\;$refers to the Kronecker delta, and *k* refers to the turbulent kinetic energy.

The eddy viscosity in this closure scheme is

(5) $$\upsilon_{t}=\frac{k}{\omega }\;$$

where the specific dissipation, *ω*, is defined as the ratio of the turbulence dissipation rate to *k*:

(6) $$\omega =\frac{\varepsilon }{\beta^{*}k}\;$$

where *ε* is the turbulent dissipation rate, and β* is the closure coefficient.

The two-dimensional *k*-*ω* steady-state transport equation is

(7) $$\rho \frac{\partial \left(U_{j}k\right)}{\partial x_{j}}=\rho \tau_{ij}\frac{\partial U_{i}}{\partial x_{j}}-\beta^{*}\rho \omega k+\frac{\partial }{\partial x_{j}}\left[\left(\mu +\mu_{t}\sigma_{k}\right)\frac{\partial k}{\partial x_{j}}\right]\;$$

(8) $$\rho \frac{\partial \left(U_{j}\omega \right)}{\partial x_{j}}=\alpha \frac{\rho \omega }{k}\tau_{ij}\frac{\partial U_{i}}{\partial x_{j}}-\beta \rho \omega^{2}+\frac{\partial }{\partial x_{j}}\left[\left(\mu +\mu_{t}\sigma_{\omega }\right)\frac{\partial \omega }{\partial x_{j}}\right]\;$$

The standard closure coefficients for the *k*-*w* scheme are obtained from Wilcox (2006):

$$\alpha =5/9,\;\beta =3/40,\;\beta *=9/100,\;\mathrm{and}\;\sigma_{k}=\sigma_{\omega }=0.5.$$

On this basis, the quadrilateral unstructured mesh is used to discretize the simulation region. The grid is refined near the sediment–water interface, the boundary layer thickness is 0.001 m, and the surface water part generates 54,000 grids with a mass of 0.97. 

#### 2.2.2. Mathematical Model and Boundary Conditions for Pore Water

In this study, it was assumed that the structure and surface flow conditions control the distribution of the water head along the riverbed surface, so the feedback of the subsurface layer was ignored. It was assumed that the head of the left and right boundary conditions corresponds to the total head of surface water at these locations, but there was no flow at the bottom boundary of the region. The boundary condition settings were shown in Figure 2. In all simulations, the sediments were homogeneous and isotropic.

The two-dimensional porous media flow in the sediment was solved by the steady groundwater flow equation:

(9) $$\frac{\partial }{\partial x_{i}}\left(-\frac{k}{\mu }\frac{\partial P}{\partial x_{i}}\right)=0\;$$

where *k* is the sediment permeability, *μ* is the fluid viscosity, and *P* is the pressure. The pressure at the sediment–water interface is determined by the surface water mathematical model.

The solute transport was modeled by the advection diffusion equation:

(10) $$\frac{\partial C}{\partial t}=\left(D_{m}+D_{ij}\right)\frac{\partial^{2}C}{\partial x_{i}{}^{2}}-\frac{q_{i}}{n}\frac{\partial C}{\partial x_{i}}\;$$

where *C* is the concentration, t is the time, n is the porosity (=0.4), and *D_m_* is the molecular diffusion coefficient in porous media. *D_ij_* is the mechanical dispersion coefficient tensor (*i*, *j* = 1, 2), which is defined as follows:

(11) $$D_{ij}=\alpha_{T}U\delta_{ij}+\frac{\left(\alpha_{L}-\alpha_{T}\right)u_{i}u_{j}}{U}\;$$

where *α_T_* and *α_L_* are the transverse and longitudinal dispersion, respectively, *U* is the pore velocity, and *δ_ij_* is the Kronecker function. The value of *α_L_* is 0.1 cm, and *α_T_* is considered to be 1/10 of *α_L_*.

For simplicity, the top boundary (SWI) was designated the concentration boundary, and the flume wall was designated the zero-flux boundary. The initial solute concentration in the sediment was 4 mol·m^−3^. Then, the simulation domain was discretized by a structured mesh with free triangular mesh elements. The mesh was refined near the SWI and the boundary wall. The bed substrate was divided into 16,600 meshes with the mass of 0.90. Table 1 summarized the parameters used in the proposed calculation model.

### 2.3. Model Evaluation

The root mean square error (RMSE), coefficient of determination (R^2^), and relative error (Re) were used to evaluate the simulation accuracy of the model.

(12) $$\mathrm{RMSE}=\sqrt{\overset{n}{\underset{i=1}{\sum }}\frac{{\left(O_{i}-S_{i}\right)}^{2}}{n}}$$

(13) $$R^{2}=1-\frac{\sum_{i=1}^{n}{\left(O_{i}-S_{i}\right)}^{2}}{\sum_{i=1}^{n}{\left(O_{i}-O\right)}^{2}}\;$$

(14) Re=∑i=1n(Oi−Si)2/∑i=1nOi2 

where *O_i_* is the measured value, *S_i_* is the model simulation value, *n* is the sample capacity, and *O* is the sample mean value.

The consistency between the measured and simulated values was measured using RMSE to verify the model. RMSE is a non-negative value, with a low RMSE indicating a good consistency between the measured and simulated values [34,35]. R^2^ is the coefficient of determination of the linear regression equation (y = x) between the measured and simulated values, and a large R^2^ indicates good consistency between the measured and simulated values [36]. Re is the relative error between the measured and simulated values, and a low Re indicates good consistency between the measured and simulated values [37].

## 3. Results

### 3.1. Model Valuation

**Figure 3:**
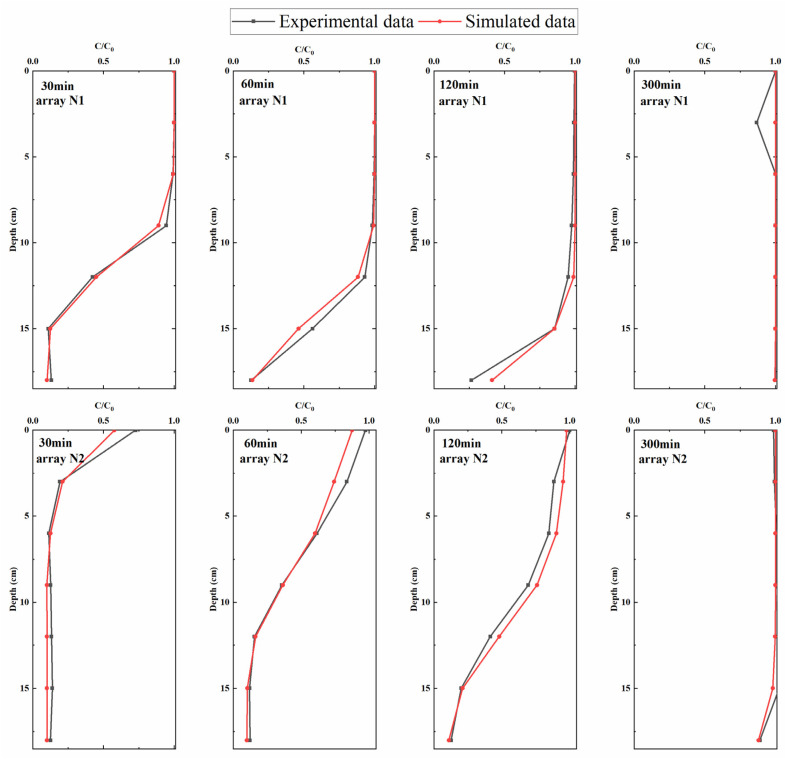
Comparison between the measured (black curves) and simulated (red curves) solute concentrations varying with depth.

The simulated sodium chloride concentration data were compared with the experimental values. The results are shown in Figure 3. It can be seen that the simulated values of typical section N1 upstream and typical section N2 downstream of the structure were in good agreement with the measured values.

Table 2 showed that the RMSEs of sections N1 and N2 were both less than 0.13, and the maximum value appeared at 120 min in section N2. The determination coefficient R^2^ was mostly greater than 0.83, and the minimum value appears at 300 min in section N1, which is 0.7522. The range of relative error Re is mostly between 1.72% and 9.22%, and the maximum value appears at 30 min in the cross-section N2, which is 19.03% and belongs to a reasonable range. In summary, the numerical simulation results obtained in this study were highly consistent with the flume test results, and the numerical model constructed could better simulate the turbulent flow of surface water and the transport of solutes in sediments.

### 3.2. Influence of Structural Proportion

To study the influence of the height ratio changes of ground and underground parts of the structure on solute transport in the hyporheic zone, five working conditions were set, as shown in Table 3. To avoid the boundary effect as much as possible, a riverbed with a length of 5 m and a depth of 1 m was set, the structure size was kept the same and located at the right center X = 2.5 m.

**Figure 4:**
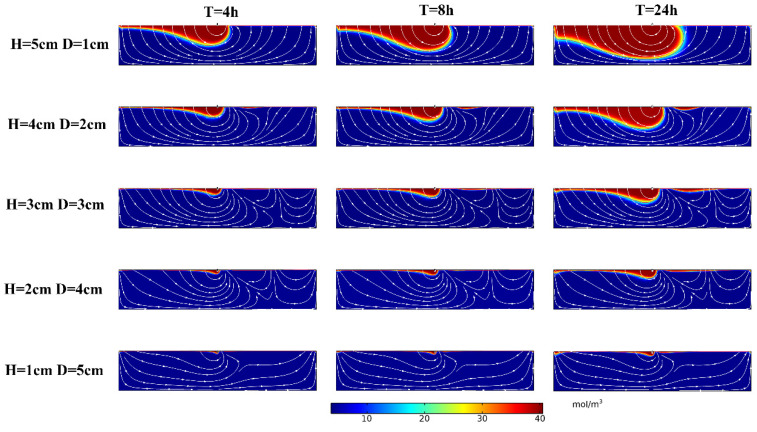
Solute transport in the hyporheic zone under the influence of structural *H* and *D* changes. The color scale for the outputs, representing solute concentration; warmer colors: higher concentration, cooler colors: lower concentration. Flow in the overlying water column (not shown) is from left to right. *T* represents time.

The simulation results are shown in Figure 4. The depth of solute transport increased with increasing time, and the solute exchange region expanded. The depth of solute transport (D_p_) increased with *H*/*D*; for example, when *T* = 8 h, the D_p_ was 0.08 m, 0.14 m, 0.22 m, 0.36 m, and 0.65 m at *H* = 1 cm, 2 cm, 3 cm, 4 cm, and 5 cm, respectively.

**Figure 5:**
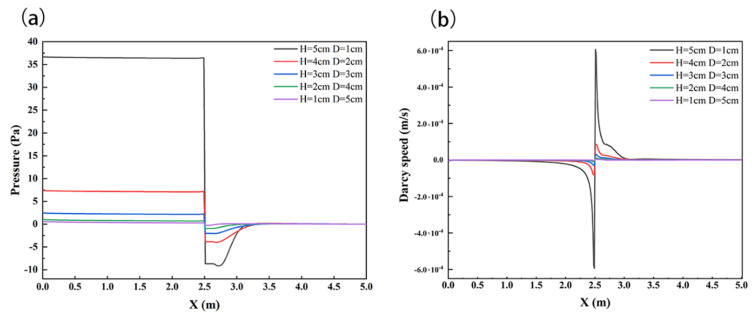
SWI pressure distribution diagram (**a**) and velocity distribution diagram (**b**) under the influence of structural *H* and *D* changes. Channel flow is left to right.

To further analyze the influence of structural position change on solute transport in the HZ, the interface pressure and velocity of sediments were analyzed, as shown in Figure 5. The pressure distribution patterns were much similar. The pressure along the SWI generally slightly decreased in the downward upstream of the weir, and it increased in the upward downstream of the weir, which was similar to the finding of Feng et al. [38]. *H*/*D* showed positive relationships with the SWI pressure; the maximum values were 0.61 Pa, 1.03 Pa, 2.48 Pa, 7.4 Pa, and 36.7 Pa at *H* = 1 cm, 2 cm, 3 cm, 4 cm, and 5 cm, respectively. At the same time, the structure significantly changed the velocity distribution on the SWI, and the velocity rate tended to increase in magnitude with the weir height *H*.

**Figure 6:**
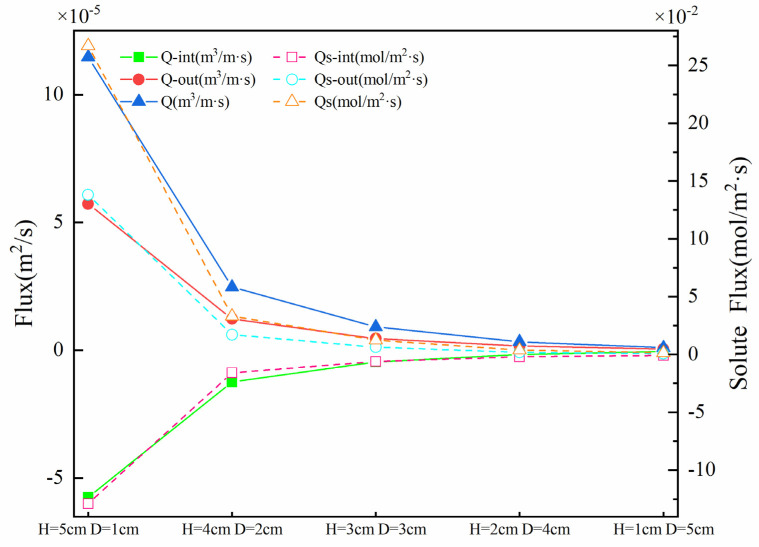
Vertical hyporheic exchange flux (Q), downwelling hyporheic flux (Q_int_), upwelling hyporheic flux (Q_out_), solute flux (Qs), downwelling solute flux (Q_s-int_), and upwelling solute flux (Q_s-out_) on the bed surface under different *H* and *D* values. The solid lines represent the hyporheic exchange flux; the dashed lines show the solute flux.

The magnitude of the vertical flux and solute flux induced by a weir could be viewed as decisive metrics of hyporheic exchange. Figure 6 shows the upwelling flux Q_out_, downwelling flux Q_int_, and SWI interface solute exchange capacity Qs under different *H* and *D*. On the whole, with the increase in *H* and the decrease in *D*, the upwelling flux (4.68 × 10^−7^~5.73 × 10^−5^ m^2^·s^−1^) and the downwelling flux (−5.20 × 10^−7^~−5.74 × 10^−5^ m^2^·s^−1^) showed an increasing trend, and the total flux Q increased with the increase in *H* (9.88 × 10^−7^~1.15 × 10^−4^ m^2^·s^−1^). Qs had a similar trend, and the total flux of solute exchange increased from 1.51 × 10^−3^ mol/(m^2^·s) to 2.67 × 10^−1^ mol/(m^2^·s). 

### 3.3. Influence of Structural Spacing

To compare the influence of different spacings (*S*) of structures on solute transport in the hyporheic zone, different values of *S* were set for numerical simulation. The position of one structure was fixed unchanged at X = 4.5 m; the other structure was set in the upper reaches of the fixed structure, and the spacing between the two structures was 0.1 m, 0.5 m, 1 m, 1.5 m, 2 m, 2.5 m, and 3 m, respectively. In Section 3.2, we found that when the height *H* = 0.05 m, the buried depth *D* = 0.01 m, the solute flux in the HZ was the largest, and the solute transport depth D_P_ was the deepest. Thus, we set *H* = 0.05 m, *D* = 0.01 m as the structure parameter. The water depth of the river was 0.06 m, and the flow velocity of the river was 0.04 m/s. The permeability *k* of the riverbed sediment also remained unchanged, at 1 × 10^−9^ m^2^.

**Figure 7:**
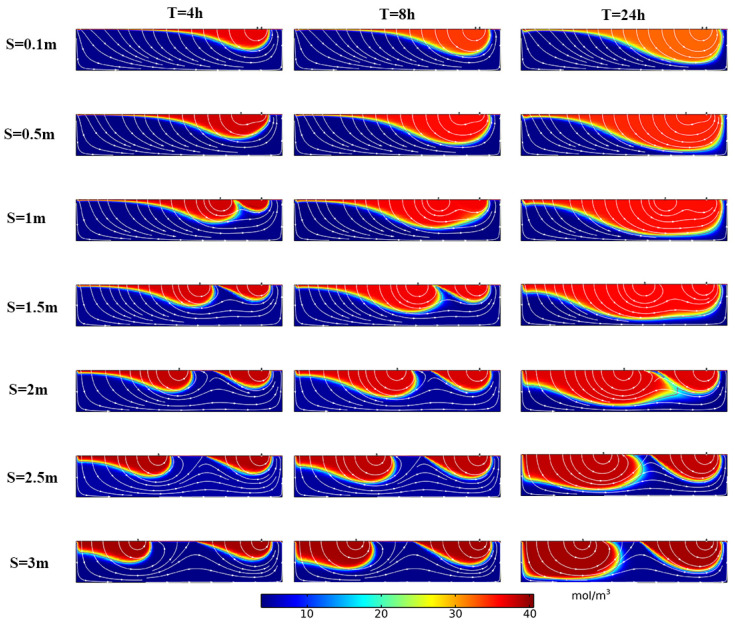
Distribution of solute concentration in the hyporheic zone under the influence of different structural spacings. *S* represents the distance between structures, *T* represents time. The color scale for the outputs, representing solute concentration; warmer colors: higher concentration, cooler colors: lower concentration. Flow in the overlying water column (not shown) is from left to right.

The simulation results are shown in Figure 7. The depth of solute transport increased with increasing time, and the solute exchange region expanded. When the T was 8 h, the depth of the solute front increased when the structure spacing *S* increased from 0.1 m to 1 m, and the D_p_ were 0.64 m, 0.72 m, 0.74 m, respectively. When the structural spacing *S* continued to increase to 1.5 m, 2 m, 2.5 m, and 3 m, the D_p_ were 0.68 m, 0.67 m, 0.66 m, and 0.68 m, respectively, indicating that when the structural spacing *S* increases, the solute front depth D_p_ increases at first and then decreases when *S* continues to increase to a certain value. 

**Figure 8:**
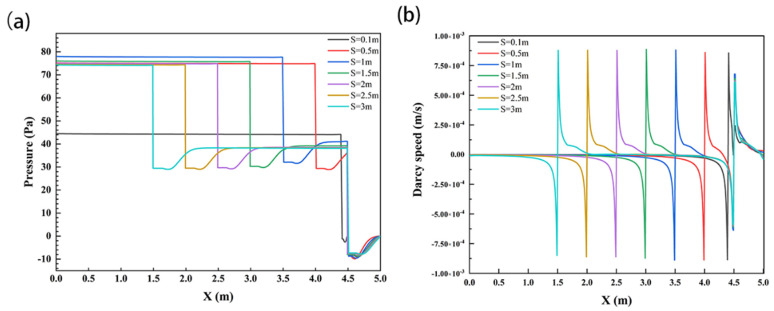
SWI pressure distribution diagram (**a**) and velocity distribution diagram (**b**) under the influence of structural spacing change. Channel flow is left to right.

To further analyze the influence of structural spacing on solute transport in the subsurface flow zone, the interface pressure and velocity of sediments were analyzed, as shown in Figure 8. The SWI pressure values of each working condition showed a slightly decreasing trend on the structure; they decreased abruptly near the structure and then increased. Comparing these working conditions, it could be found that the pressure value first increased and then decreased with increasing structural spacing. The maximum values were 44.55 Pa, 75.26 Pa, 78.04 Pa, 76.1 Pa, 75.1 Pa, 74.6 Pa, and 74.3 Pa at *S* = 0.1 m, 0.5 m, 1 m, 1.5 m, 2 m, 2.5 m, and 3 m, respectively. At the same time, the structure significantly changed the velocity distribution on the SWI, but the maximum velocity value did not change significantly with the increase in *S*, all of which were approximately 8.8 × 10^−4^ m/s.

**Figure 9:**
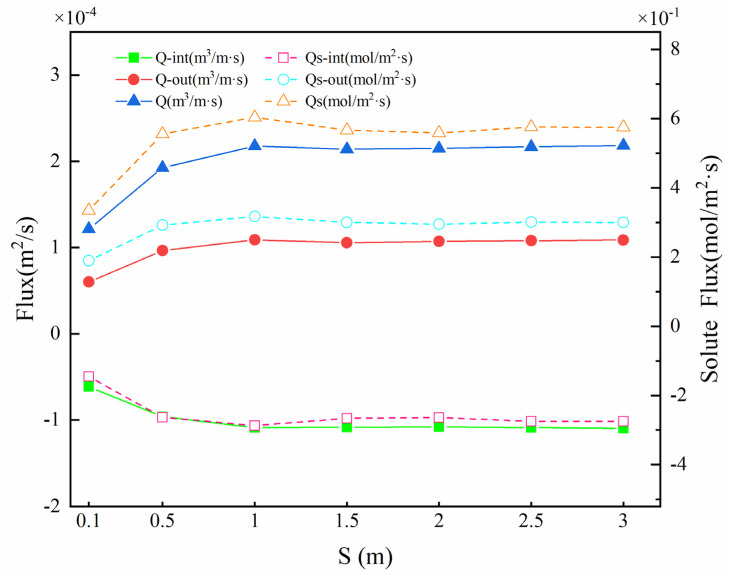
Vertical hyporheic exchange flux (Q), downwelling hyporheic flux (Q_int_), upwelling hyporheic flux (Q_out_), solute flux (Qs), downwelling solute flux (Q_s-int_), and upwelling solute flux (Q_s-out_) under different *S* values. The solid lines represent the hyporheic exchange flux; the dashed lines show the solute flux.

Figure 9 shows the upwelling flux Q_out_, downwelling flux Q_int_, and SWI interface solute exchange capacity Qs under different *S*. On the whole, with the increase in structural spacing *S*, the upwelling flux (6.01 × 10^−5^~1.09 × 10^−4^ m^2^·s^−1^) and the downward flux (−6.15 × 10^−5^~−1.10 × 10^−4^ m^2^·s^−1^) first increased and then decreased, and the total flux Q first increased and then remained unchanged (1.22 × 10^−4^~2.18 × 10^−4^ m^2^·s^−1^). The trend of Qs was different; the total flux of solute exchange increased from 3.35 × 10^−1^ mol/(m^2^·s) to 6.04 × 10^−1^ mol/(m^2^·s) and then decreased to 5.59 × 10^−1^ mol/(m^2^·s). 

### 3.4. Influence of Structural Number

To compare the influence of the number of structures (*N*) on solute transport in the river HZ, different numbers of structures were set for numerical simulation. The number of structures was *N* = 1, 2, 3, and 4. In Section 3.3, we found that the solute exchange flux Qs and solute front depth D_p_ were the largest when *S* = 1 m, so the distance *S* between each structure was fixed at 1 m. The position of a fixed structure was unchanged at X = 4.5 m, and other structures were arranged in the upstream area of the fixed structure. The structure height *H* was 0.05 m, and the buried depth *D* = 0.01 m remained unchanged. The surface water depth was 0.06 m, the velocity was 0.04 m/s, and the permeability *k* of the bed bottom material was 1 × 10^−9^ m^2^.

**Figure 10:**
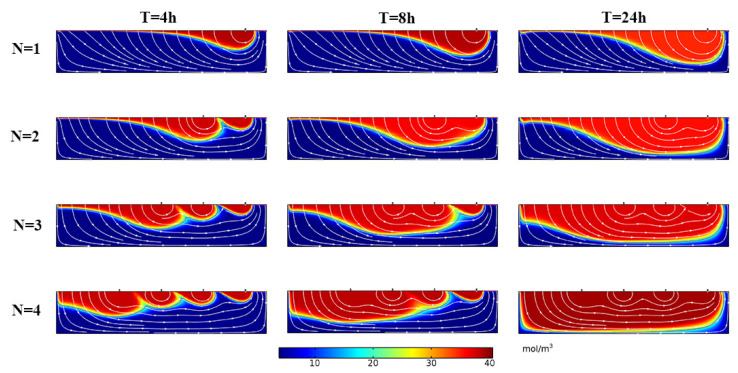
The distribution of solute concentration in the hyporheic zone under the influence of different structure numbers *N*. The color scale for the outputs, representing solute concentration; warmer colors: higher concentration, cooler colors: lower concentration. Flow in the overlying water column (not shown) is from left to right. *T* represents time.

The simulation results are shown in Figure 10. The solute transport depth D_p_ increased with increasing time, and the solute exchange region increased obviously. By comparing the conditions of different numbers, it could be seen that the D_p_ increased with the structure number *N*; for example, when *T* = 8 h, the D_p_ was 0.6 m, 0.7 m, 0.73 m, and 0.77 m at *N* = 1, 2, 3, and 4, respectively, which indicated that increasing the number of structures could significantly increase the solute exchange depth in the HZ and expand the exchange range.

**Figure 11:**
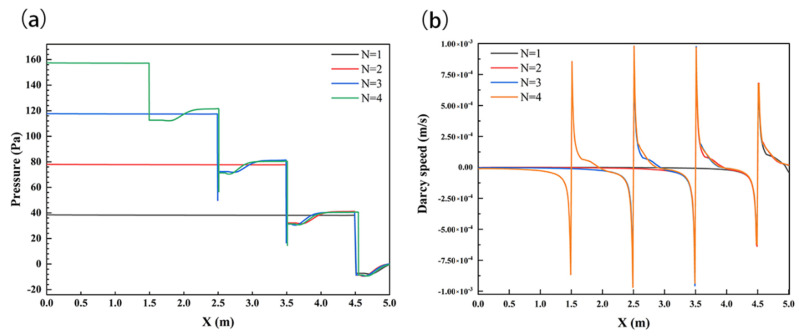
SWI pressure distribution (**a**) and velocity distribution (**b**) under the influence of different structure numbers *N*. Channel flow is left to right.

To further analyze the influence of the number of structures on the solute transport in the subsurface, the pressure and velocity of the sediment interface were analyzed, as shown in Figure 11. *N* showed positive relationships with the SWI pressure; the maximum values were 38.5 Pa, 78 Pa, 118 Pa, and 157 Pa at *N* = 1, 2, 3, and 4, respectively. At the same time, the setting of the structure significantly changed the flow velocity distribution on the SWI, and the velocity increased with the number of structures; the maximum velocity values were 6.09 × 10^−4^ m/s, 8.88 × 10^−4^ m/s, 9.75 × 10^−4^ m/s, and 9.79 × 10^−4^ m/s at *N* = 1, 2, 3, and 4, respectively.

**Figure 12:**
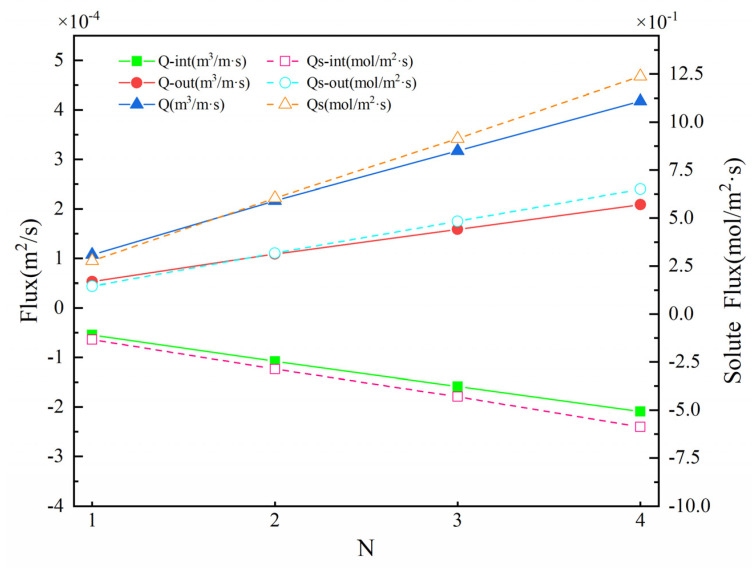
Vertical hyporheic exchange flux (Q), downwelling hyporheic flux (Q_int_), upwelling hyporheic flux (Q_out_), solute flux (Qs), downwelling solute flux (Q_s-int_), and upwelling solute flux (Q_s-out_) under different *N* values. The solid lines represent the hyporheic exchange flux; the dashed lines show the solute flux.

The hyporheic exchange flux under the influence of the number of structures was analyzed. Figure 12 shows the upwelling flux Q_out_, downwelling flux Q_int_, and SWI interface solute exchange capacity Qs under different *N*. On the whole, with the increase in the number, the upwelling flux (5.34 × 10^−5^~2.09 × 10^−4^ m^2^·s^−1^), downwelling flux (−5.42 × 10^−5^~−2.09 × 10^−4^ m^2^·s^−1^), and Q increased (1.08 × 10^−4^–4.18 × 10^−4^ m^2^·s^−1^). Qs had a similar trend, and the total flux of solute exchange increased from 2.77 × 10^−1^ m^2^·s^−1^ to 1.24 mol/(m^2^·s). This showed that the number of structures *N* had a positive correlation with Q and Qs. The increase in *N* significantly promoted solute transport in the hyporheic zone and increased the hyporheic exchange magnitude.

## 4. Discussion

### 4.1. Influence of Structural Design Parameters

The arrangement of the structure could enhance the hyporheic exchange of the riverbed, but different design parameters of the structure have different influences on the hyporheic exchange. To our knowledge, there are few studies on the influence of *H*/*D*, spacing change, and number change on hyporheic exchange. Our numerical simulations suggested that the vertical water exchange flux Q, solute exchange flux Qs, and solute transport depth D_p_ increased with *H*/*D* and number (*N*), while Q, D_p_, and Qs increased with the structural spacing (*S*) to begin with; then, Q remained constant, and D_p_ and Qs decreased as *S* continued to increase.

In fact, this research indicated that the height of the structure above the ground (*H*) was more important than its height below the ground (*D*), and the hyporheic exchange flux Q was positively correlated with *H*, which was consistent with Hester and Doyle [30], whose research showed that the downwelling flux was linearly correlated with the structure size. In fact, in this study, when the height *H* of the structure changed, the permeability *k* of the sediment remained unchanged, so Darcy’s law can be simplified as follows:

(15) $$Q=k\frac{\Delta h}{l}A$$

where *A* is the cross-sectional area of the hyporheic path, and Δ*h* is the in-stream head drop across the structure. The relationship among Q, Qs, and structure height *H* is consistent with the changing law of the relationship between pressure on the SWI and *H*. Therefore, it is clear that Δh in Equation (15) is more important than *A* or *l* in determining Q and that the in-stream structure controls Q primarily by controlling the interface pressure drop. In general, the magnitudes of Q and Qs are positively correlated with the height *H* of the structure, and the structure mainly controls Q by controlling the increase in *H*, which promotes solute transport and exchange in the hyporheic zone. Similarly, in our research, there is a positive correlation between the number of structures *N* and Q, Qs. The increase in the number of structures *N* is mainly to enhance the hyporheic exchange by increasing the Δ*h* of this reach and then to increase the Q.

It is worth noting that the hyporheic flux and solute transport under the influence of structural spacing *S* are different from *H* and *N*. In our study, Q, D_P_, and Qs increased with *S* to begin with; then, Q remained constant, and D_P_ and Qs decreased as *S* continued to increase. Based on the analysis of the pressure distribution, it was found that the pressure value of SWI also showed the same rule, and the maximum pressure reached the highest when *S* = 1 m. We compared the solute flux (0.575 mol/(m^2^·s)) at *S* = 3 m with the solute flux at *N* = 1 (0.277 mol/(m^2^·s)) and found that the solute flux Qs at *S* = 3 m was close to twice the solute flux at *N* = 1, which indicated that the coupling relationship between the two structures gradually disappeared when the structure spacing *S* continued to increase, and these two structures each functioned independently. Of course, the optimal solution of the *S* value needs further research.

Of course, this model still has certain limitations. However, we expanded the length and depth of the model to avoid the boundary effect as much as possible. This effect is acceptable, and we would solve this problem in subsequent studies.

### 4.2. Implications for River Restoration Design

Weirs are common structures in river restoration projects, which are designed to enhance natural features, such as pools and shoals, increase biophysical heterogeneity, and provide habitat for fish. Our research showed that the setting of the structure in the channel could indeed improve the hyporheic exchange and solute transport, and the height of the structure had a greater impact on the hyporheic exchange and solute exchange, which was consistent with the results of Ward, Gooseff, and Johnson [31]. They obtained a sensitivity analysis in which the structure size, especially the structure height, was the most important influencing parameter. With increasing weir height, the potential circulation increased linearly. Secondly, the influence of structure number on the hyporheic exchange was also an important parameter. In this study, we found that with the increase in structure number *N*, hyporheic exchange flux Q also presented a linear increase, which showed that the river ecological engineering restoration technology effectively increased the river hyporheic exchange strength and improved the exchange flux. Therefore, in the river ecological restoration project, we could increase the height and number of the structure within a certain river range to provide a larger hyporheic flow. Of course, the spacing of structures must be considered when increasing the number of structures. We found that a smaller spacing and larger spacing had less effect on improving the latent flow flux. Therefore, we should choose a more suitable spacing to set up the restoration structure to better play the role of the structure.

More importantly, in actual engineering, the actual situation near the river must be investigated to determine whether the structure of the recovery effect largely depends on the surrounding groundwater discharge and supply efficiency, riverbed permeability, and surface water flow rate. This will be our next research focus, as the actual river engineering structure can play an effective role. Of course, the influence of structure on the temperature and residence time of the HZ is also an important parameter to be considered. Temperature is related to the living environment of microorganisms in the river HZ, which is closely related to the quality of river habitat. The residence time is related to the biogeochemical cycle process in the HZ, and the residence time required can be obtained through structural design to make the desired biogeochemical reaction occur [25,39].

## 5. Summary and Conclusions

Structural design parameters change is an important factor affecting hyporheic exchange and solute transport. In this study, the data of the flume experiment were used for verification, and COMSOL Multiphysics numerical simulation software was used to simulate the structure of different design parameters, mainly including the structure position, number, and spacing. The main conclusions are as follows.

The accuracy of the model was verified by the data of the indoor flume experiment using the RMSE, R^2^, and Re as evaluation indices. In summary, the numerical simulation results obtained in this paper were highly consistent with the flume test results, and the numerical model constructed could better simulate the turbulent flow of surface water and the transport of solutes in sediments.

The single-factor effects of *H*/*D*, *S* and *N* on hyporheic exchange were investigated by numerical simulations. The results showed that the depth of solute transport (D_p_), vertical hyporheic exchange flux (Q), and solute flux (Qs) increased with the structural height *H* and number *N.* The structure spacing *S* had a positive correlation with the hyporheic exchange in a certain range. Q, D_p_, and Qs increased with the structural spacing (*S*) to begin with, and then Q remained constant. D_p_ and Qs decreased as *S* continued to increase.

Generally, the *H*/*D* (especially the height above the sediment *H*), number, and spacing of structures are important design parameters in ecological restoration projects of the hyporheic zone, and the influence mechanism of these parameters on hyporheic exchange and solute transport is still an important issue for future research. Follow-up work should still discuss the quantitative relationship between the number and spacing of structures and other indices of hyporheic exchange (such as residence time) and the influence degree of structures on solute transport under different surface water velocities and sediment permeabilities, which is of great significance for determining the design indices of engineering structures.

## Figures and Tables

**Table 1:** Parameter settings of the numerical simulation experiment.

Surface Water Velocity (m/s)	Porosity Ratio *n*	Permeability *k* (m^2^)	Fluid Viscosity *μ* (Pa·s)	Molecular Diffusion Coefficient *D_m_* (m^2^/s)
0.04	0.4	1 × 10^−9^	0.0011	5 × 10^−10^

**Table 2:** Accuracy of the vertical simulation of solute concentrations at different monitoring points and periods.

	Array N1			Array N2		
Time	RMSE	R^2^	Re%	RMSE	R^2^	Re%
30 min	0.0263	0.9955	7.10%	0.0617	0.9119	19.03%
60 min	0.0417	0.9822	4.42%	0.0527	0.9745	9.16%
120 min	0.0583	0.9444	9.22%	0.1291	0.8392	8.45%
300 min	0.0527	0.8987	3.48%	0.0218	0.7522	1.72%

**Table 3:** Experimental parameter table of structural proportion change.

**Case**	**u_0_**	**Water Depth**	**Width**	**Depth (*D*)**	**Height (*H*)**	***H***/***D***
1	4 cm/s	6 cm	10 cm	5 cm	1 cm	1/5
2	4 cm/s	6 cm	10 cm	4 cm	2 cm	2/4
3	4 cm/s	6 cm	10 cm	3 cm	3 cm	3/3
4	4 cm/s	6 cm	10 cm	2 cm	4 cm	4/2
5	4 cm/s	6 cm	10 cm	1 cm	5 cm	5/1

## Data Availability

The data presented in this study are available on request from the corresponding author. The data are not publicly available due to the subsequent studies will be conducted on this basis.

## References

[1] Hester E.T., Gooseff M.N. (2010). Moving Beyond the Banks: Hyporheic Restoration Is Fundamental to Restoring Ecological Services and Functions of Streams. Environ. Sci. Technol..

[2] Boulton A.J., Findlay S., Marmonier P., Stanley E.H., Valett H.M. (1998). The Functional Significance of the Hyporheic Zone in Streams and Rivers. Annu. Rev. Ecol. Syst..

[3] Liu S., Chui T.F.M. (2017). Impacts of Streambed Heterogeneity and Anisotropy on Residence Time of Hyporheic Zone. Groundwater.

[4] Peterson E.W., Sickbert T.B., Moore S.L. (2008). High frequency stream bed mobility of a low-gradient agricultural stream with implications on the hyporheic zone. Hydrol. Process..

[5] Peralta-Maraver I., Reiss J., Robertson A.L. (2018). Interplay of hydrology, community ecology and pollutant attenuation in the hyporheic zone. Sci. Total Environ..

[6] Liu S., Chui T.F.M. (2020). Optimal In-Stream Structure Design through Considering Nitrogen Removal in Hyporheic Zone. Water.

[7] Krause S., Hannah D.M., Fleckenstein J.H. (2009). Hyporheic hydrology: Interactions at the groundwater-surface water interface. Hydrol. Process..

[8] Huettel M., Ziebis W., Forster S. (1996). Flow-Induced Uptake of Particulate Matter in Permeable Sediments. Limnol. Oceanogr..

[9] Hester E.T., Cardenas M.B., Haggerty R., Apte S.V. (2017). The importance and challenge of hyporheic mixing. Water Resour. Res..

[10] Swanson E.T., Cardenas M.B. (2010). Diel Heat Transport within the Hyporheic Zone of a Pool-Riffle-Pool Sequence of a Losing Stream and Evaluation of Models for Fluid Flux Estimation Using Heat. Limnol. Oceanogr..

[11] Norman F.A., Cardenas M.B. (2014). Heat transport in hyporheic zones due to bedforms: An experimental study. Water Resour. Res..

[12] Packman A.I., Salehin M., Zaramella M. (2004). Hyporheic Exchange with Gravel Beds: Basic Hydrodynamic Interactions and Bedform-Induced Advective Flows. J. Hydraul. Eng..

[13] Packman A.I., Brooks N.H., Morgan J.J. (2000). A physicochemical model for colloid exchange between a stream and a sand streambed with bed forms. Water Resour. Res..

[14] Roche K.R., Li A., Bolster D., Wagner G.J., Packman A.I. (2019). Effects of Turbulent Hyporheic Mixing on Reach-Scale Transport. Water Resour. Res..

[15] Soulsby C., Malcolm I., Youngson A. (2001). Hydrochemistry of the hyporheic zone in salmon spawning gravels: A preliminary assessment in a degraded agricultural stream. Regul. Rivers Res. Manag..

[16] Groffman P.M., Dorsey A.M., Mayer P.M. (2005). N processing within geomorphic structures in urban streams. J. N. Am. Benthol. Soc..

[17] Harvey J., Gooseff M. (2015). River corridor science: Hydrologic exchange and ecological consequences from bedforms to basins. Water Resour. Res..

[18] Lewandowski J., Arnon S., Banks E., Batelaan O., Betterle A., Broecker T., Coll C., Drummond J.D., Garcia J.G., Galloway J. (2019). Is the Hyporheic Zone Relevant Beyond the Scientific Community?. Water.

[19] Hancock P.J., Boulton A.J., Humphreys W. (2005). Aquifers and hyporheic zones: Towards an ecological understanding of groundwater. Appl. Hydrogeol..

[20] Boulton A.J. (2007). Hyporheic rehabilitation in rivers: Restoring vertical connectivity. Freshw. Biol..

[21] Boulton A.J., Datry T., Kasahara T., Mutz M., Stanford J.A. (2010). Ecology and management of the hyporheic zone: Stream–groundwater interactions of running waters and their floodplains. J. N. Am. Benthol. Soc..

[22] Hester E.T., Brooks K.E., Scott D.T. (2018). Comparing reach scale hyporheic exchange and denitrification induced by instream restoration structures and natural streambed morphology. Ecol. Eng..

[23] Kasahara T., Wondzell S.M. (2003). Geomorphic controls on hyporheic exchange flow in mountain streams. Water Resour. Res..

[24] Mutz M., Kalbus E., Meinecke S. (2007). Effect of instream wood on vertical water flux in low-energy sand bed flume experiments. Water Resour. Res..

[25] Smidt S., Cullin J.A., Ward A.S., Robinson J., Zimmer M.A., Lautz L.K., Endreny T.A. (2014). A Comparison of Hyporheic Transport at a Cross-Vane Structure and Natural Riffle. Groundwater.

[26] Dudunake T., Tonina D., Reeder W.J., Monsalve A. (2020). Local and Reach-Scale Hyporheic Flow Response from Boulder-Induced Geomorphic Changes. Water Resour. Res..

[27] Rana S.M., Scott D.T., Hester E.T. (2017). Effects of in-stream structures and channel flow rate variation on transient storage. J. Hydrol..

[28] Hester E.T., Hammond B., Scott D.T. (2016). Effects of inset floodplains and hyporheic exchange induced by in-stream structures on nitrate removal in a headwater stream. Ecol. Eng..

[29] Wade J., Lautz L., Kelleher C., Vidon P., Davis J., Beltran J., Pearce C. (2020). Beaver dam analogues drive heterogeneous groundwater–surface water interactions. Hydrol. Process..

[30] Hester E.T., Doyle M.W. (2008). In-stream geomorphic structures as drivers of hyporheic exchange. Water Resour. Res..

[31] Ward A.S., Gooseff M.N., Johnson P.A. (2011). How Can Subsurface Modifications to Hydraulic Conductivity Be Designed as Stream Restoration Structures? Analysis of Vaux’s Conceptual Models to Enhance Hyporheic Exchange. Water Resour. Res..

[32] Jin G., Tang H., Gibbes B., Li L., Barry D. (2010). Transport of nonsorbing solutes in a streambed with periodic bedforms. Adv. Water Resour..

[33] Cardenas M.B., Wilson J. (2007). Hydrodynamics of coupled flow above and below a sediment–water interface with triangular bedforms. Adv. Water Resour..

[34] Mentaschi L., Besio G., Cassola F., Mazzino A. (2013). Problems in RMSE-based wave model validations. Ocean Model..

[35] Ren J., Wang X., Zhou Y., Chen B., Men L. (2019). An Analysis of the Factors Affecting Hyporheic Exchange based on Numerical Modeling. Water.

[36] Quinino R.C., Reis E.A., Bessegato L.F. (2013). Using the Coefficient of Determination R2 to Test the Significance of Multiple Linear Regression. Teach. Stat..

[37] Suñé V., Carrasco J.A. (2005). Efficient implementations of the randomization method with control of the relative error. Comput. Oper. Res..

[38] Feng J., Liu D., Liu Y., Li Y., Li H., Chen L., Xiao J., Liu J., Dong J. (2022). Hyporheic exchange due to in-stream geomorphic structures. J. Freshw. Ecol..

[39] Ward B.B., Devol A.H., Rich J.J., Chang B.X., Bulow S.E., Naik H., Pratihary A., Jayakumar A. (2009). Denitrification as the dominant nitrogen loss process in the Arabian Sea. Nature.

